# (How) does 1,3,5-triethylbenzene scaffolding work? Analyzing the abilities of 1,3,5-triethylbenzene- and 1,3,5-trimethylbenzene-based scaffolds to preorganize the binding elements of supramolecular hosts and to improve binding of targets

**DOI:** 10.3762/bjoc.8.1

**Published:** 2012-01-02

**Authors:** Xing Wang, Fraser Hof

**Affiliations:** 1 University of Victoria, Department of Chemistry, Victoria, BC, V8W 3V6, Canada

**Keywords:** binding affinity, entropy, molecular recognition, scaffolds, supramolecular hosts, triethylbenzene, trimethylbenzene

## Abstract

1,3,5-triethylbenzenes have been widely used as supramolecular templates to organize molecular-recognition elements. It is believed that the steric-gearing effect of the 1,3,5-triethylbenzene template directs the binding elements toward the same face of the central ring, hence increasing the binding affinity. At the same time the 1,3,5-trimethylbenzene scaffold, without steric-gearing effects, has also been found to improve the binding affinities of hosts compared to the unsubstituted analogues. By studying experimental data from the literature and the Cambridge Structural Database, as well as by conducting computational studies of representative structures, we concluded that the steric gearing offered by the ethyl groups confers some energetic advantage over the methyl groups, but the size of this advantage can be small and is dependent on the groups involved.

## Introduction

**Figure 1:**
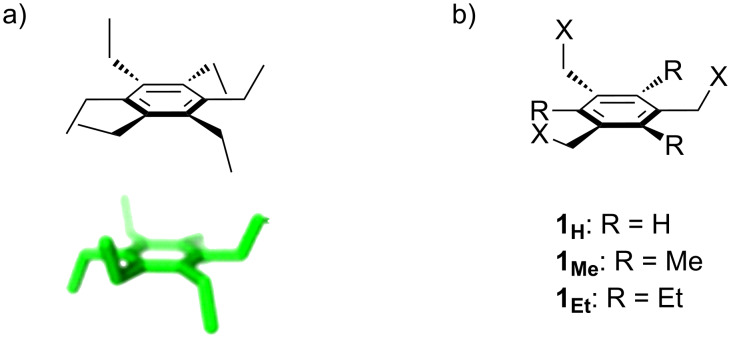
a) The global minimum-energy conformation for hexaethylbenzene reveals the basis for steric gearing in crowded arenes. b) A generalized set of hosts based on 1,3,5-triethylbenzene (**1_Et_)**, 1,3,5-trimethylbenzene (**1_Me_)**, and an unsubstituted analogue (**1_H_)**.

**Figure 2:**
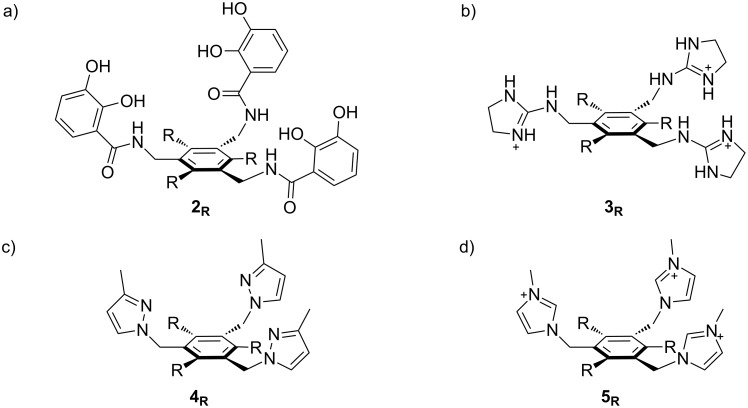
Structures of hosts discussed in this manuscript. R = H, Me, or Et.

Supramolecular hosts use arrays of multiple weak interactions to achieve strong and specific binding to targeted guest molecules. Many important weak interactions are directional and lead to highly ordered host–guest complexes [1–2]. The preorganization of binding elements in a competent binding geometry can have enthalpic effects on binding when considering the energy that must be paid to adopt a (potentially unfavorable) binding conformation, and can also have effects on binding entropy when one considers the degrees of freedom in the host, guest, and solvated host–guest complex. Rigid macrocyclic hosts are often successful because of their high degree of preorganization [3–4]. As a nonmacrocyclic alternative, 1,3,5-triethylbenzenes are widely used as an easy-to-synthesize and general scaffold for presenting molecular-recognition elements in a convergent manner (Figure 1) [1,5]. These systems were spawned by the work of Mislow, who studied the conformational preferences of hexaethylbenzene by calculation, NMR, and crystallography and concluded that the conformation bearing alternating up-down arrangement of substituents was the global minimum for this system (Figure 1) [6]. This preference arises from steric gearing of adjacent substituents, which are positioned to be as far from their respective neighbors as possible. Even before Mislow’s systematic study on this topic, Wilson and co-workers obtained the crystal structure of hexa(thiophenyl)benzene. The six thiophenyl groups are arranged around the central ring in alternating up–down fashion [7]. Raymond first took advantage of this kind of steric gearing by leaving ethyl groups in positions 1,3,5 and replacing the substituents in positions 2,4,6 with metal-coordinating ligands, which by design were directed toward the same face of the central scaffold and therefore preorganized for metal chelation (Figure 2a) [8]. The field has since exploded, with the first all-organic host–guest system of this type constructed by Anslyn [9] (Figure 2b) and over 150 papers reporting on 900 such structures for binding organic and inorganic guests having been published in the last 30 years [10]. The preorganizing effect of 1,3,5-triethylbenzene-based hosts (**1_Et_**) has generally been demonstrated by comparing them to analogues that are unsubstituted at the 1,3,5 positions (**1_H_**). But a parallel set of literature reports describes structures (**1_Me_**) based on the 1,3,5-trimethylbenzene scaffold (700 structures in 300 papers identified by SciFinder substructure searches) [10]. These have no basis for producing the steric gearing that would favor a convergent conformation of binding elements, but their binding affinities can also be improved relative to analogous unsubstituted systems (**1_H_**). The direct comparison of the 1,3,5-triethylbenzene (**1_Et_**) and 1,3,5-trimethylbenzene (**1_Me_**) templates in a single system is very rare (see below), which raises some questions: To what extent do ethyl substituents improve the binding properties of a host? To what extent do methyl substituents improve the binding properties of a host? What evidence exists for different enthalpic and entropic effects that might be responsible for the observed binding data in these families of hosts? In this paper, we report on our efforts to answer these questions using experimental data mined from the literature and from the Cambridge Structural Database, as well as with computational analysis of some representative host systems. We hope that these simple computational approaches might be more broadly useful for predicting the behavior of new supramolecular hosts.

## Results and Discussion

### Literature binding affinities

Chelating ligand **2_Et_**, the forerunner of all hosts in this class, displayed *K*_assoc_ = 10^47^ M^−1^ for Fe, which is 10^4^ or 5.4 kcal/mol stronger binding than the control host **2**_H_ (*K*_assoc_ = 10^43^ M^−1^) [8]. Anslyn’s host **3_Et_**, a host that does not rely on strong metal–ligand interactions, binds citrate only 0.6 kcal/mol more strongly than its congener **3_H_** [9]. One other host in this class that we were able to track down in the literature for direct comparisons of ethyl-substituted and unsubstituted hosts gives ∆∆*G* = 2.3 kcal/mol (Table 1, entry 1). These values provide a mixed picture of the impact of ethyl substitution.

**Table 1:** Affinity comparisons of ethyl-substituted and unsubstituted hosts.

Entry [ref.]	R group	Guest	K_assoc_ values (M^−1^)	∆∆*G*^a^ (kcal/mol)

1 [11]	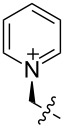	Br^−^	**6_Et_** = 8.5 × 10^2^ **6_H_** = 17	2.3
2 [8]	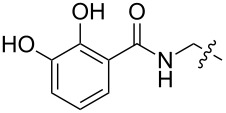	Fe	**7_Et_** = 10^47^ **7_H_** = 10^43^	5.4
3 [9]	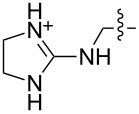	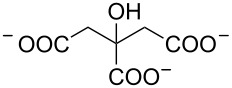	**8_Et_** = 6.9 × 10^3^ **8_H_** = 2.4 × 10^3^	0.6

^a^∆∆*G* calculated from differences in reported *K*_assoc_ values. We estimate the errors as ±20%, depending on the measuring technique used in the literature.

A separate summary of comparisons of methyl-substituted and unsubstituted hosts is presented in Table 2. The average ∆∆*G* is 0.3 kcal/mol, with a maximum of 2.3 kcal/mol and a minimum of −0.8 kcal/mol.

**Table 2:** Affinity comparisons of methyl-substituted and unsubstituted hosts.

Entry [ref.]	R group	Guest	*K*_assoc_ values (M^−1^)	∆∆*G*^a^ (kcal/mol)

1 [12]	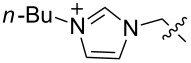	Cl^−^	**9_Me_** = 7.5 × 10^4^ **9_H_** = 1.5 × 10^3^	2.3
2 [13]	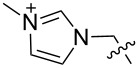	H_2_PO_4_^−^	**10_Me_** = 2.1 × 10^3^ **10_H_** = 2.0 × 10^3^	0.04
		HSO_4_^−^	**10_Me_** = 1.1 × 10^3^ **10_H_** = 1.2 × 10^3^	−0.05
		Cl^−^	**10_Me_** = 1.1 × 10^3^ **10_H_** = 1.0 × 10^3^	0.04
		Br^−^	**10_Me_** = 1.8 × 10^2^ **10_H_** = 7.6 × 10^2^	−0.8

^a^∆∆*G* calculated from differences in reported *K*_assoc_ values. We estimate the errors as ±20%, depending on the measuring technique used in the literature.

So is the ethyl substitution better than methyl? We found four papers that directly reported the binding affinities of seven different 1,3,5-triethylbenzene-based and analogous 1,3,5-trimethylbenzene-based tripodal hosts for their respective guests. These results are reported in Table 3. From this limited amount of literature data we see a range of binding-affinity differences for ethyl- and methyl-substituted hosts, which range from no difference to a 17-fold difference, with an average ∆∆*G* of 0.4 kcal/mol in favor of ethyl substitution.

**Table 3:** Direct affinity comparisons of ethyl- and methyl-substituted hosts.

Entry [ref.]	R group	Guest	*K*_assoc_ values (M^−1^)	∆∆*G*^a^ (kcal/mol)

1 [14]	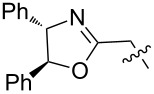	*n-*BuNH_3_^+^	**11_Et_** = 5.9 × 10^7^ **11_Me_** = 2.0 ×10^6^	2.0
		*sec-*BuNH_3_^+^	**11_Et_** = 8.3 × 10^5^ **11_Me_**= 8.3 × 10^4^	1.4
		*t-*BuNH_3_^+^	**11_Et_** = 1.5 × 10^4^ **11_Me_** = 4.7 × 10^3^	0.7
2 [15]	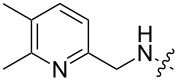	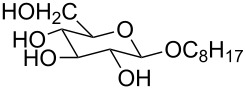	**12_Et_** = 48630 **12_Me_** = 20950	0.5
		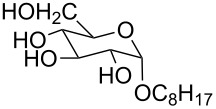	**12_Et_** = 1310 **12_Me_** = 800	0.3
3 [15]	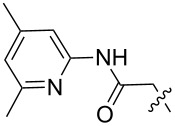	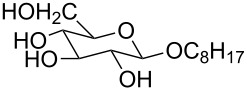	**13_Et_** = 1230 **13_Me_** = 650	0.4
5 [15]	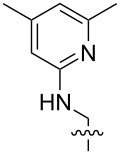	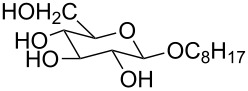	**14_Et_** = 48630 **14_Me_** = 20950	0.5
		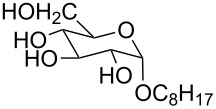	**14_Et_** = 1310 **14_Me_** = 800	0.3
		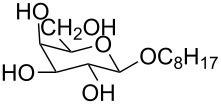	**14_Et_** = 3070 **14_Me_** = 1360	0.5
6 [15]	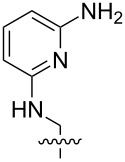	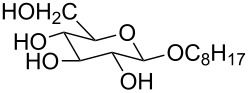	**15_Et_** = 19590 **15_Me_** = 9500	0.4
		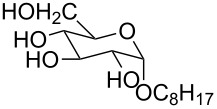	**15_Et_** = 1100 **15_Me_** = 620	0.3
7 [15]	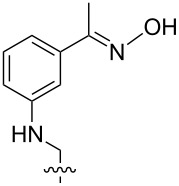	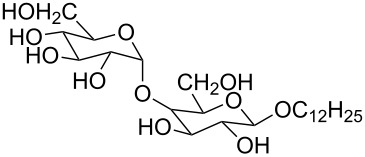	**16_Et_** = 98900 **16_Me_** = 96300	0.02
		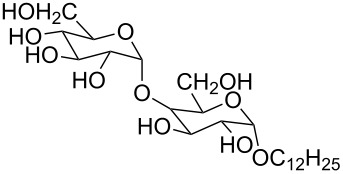	**16_Et_** = 58600 **16_Me_** = 62000	0.03
8 [16]	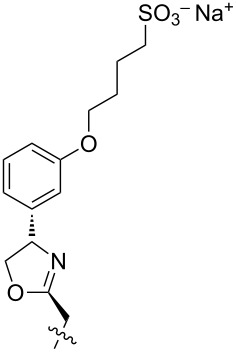	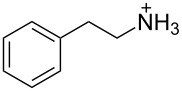	**17_Et_** = 86 **17_Me_** = 82	0.03
		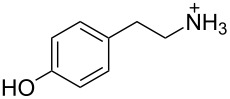	**17_Et_** = 101 **17_Me_** = 92	0.06
		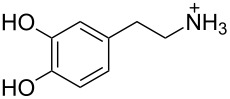	**17_Et_** = 178 **17_Me_** = 161	0.06
		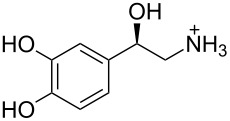	**17_Et_** = 74 **17_Me_** = 67	0.06
		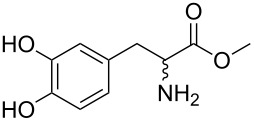	**17_Et_** = 72 **17_Me_** = 65	0.06

^a^∆∆*G* calculated from differences in reported *K*_assoc_ values. We estimate the errors as ±20%, depending on the measuring technique used in the literature.

### Crystallographic analysis of conformations

**Figure 3:**
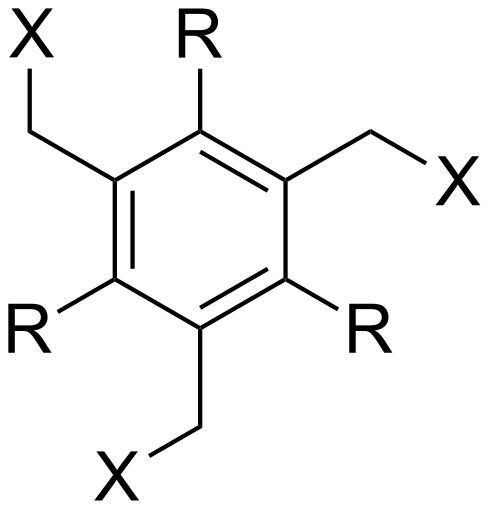
Generalized structural fragments used for mining the Cambridge Structure Database. R = Me and Et, X = N, O, C, Br.

We next carried out a survey of the literature data in the Cambridge Structure Database to evaluate the conformations adopted by 1,3,5-triethylbenzene- and 1,3,5-trimethylbenzene-derived hosts in the solid state. One must always be cautious in interpreting crystallographic data on molecular conformations, as it is subject to crystal packing influences, which are not present in solution. However, those artifacts are diminished in surveys that contain many structures, making this a generally reliable way to get a qualitative overview of a class of functional groups. We first used generalized triethylbenzene substructures (Figure 3) to retrieve records for all related organic molecules, and discarded from our analysis those whose conformations were predetermined by macrocyclizations (and that therefore were not under the control of steric gearing). In total, 126 such crystal structures of tripodal triethylbenzene-based hosts were found in the database. Among these, 86 (68.3%) were in the up–down alternating conformation in which all ethyl groups are on one face of the central ring and all binding elements are on the other, while the remainder showed some deviation from this ideal. It is also interesting to note in this section that in Mislow’s original reports on hexaethylbenzene, the presence of η^6^-coordinated Mo(CO)_3_ or Cr(CO)_3_ produced crystal structures, showing that the bound metal did not perturb the predicted up–down alternating conformation [6]. The coordination of the larger Cr(CO)_2_PPh_3_ fragment produced instead a crystal structure in which the highly unfavorable all-up conformation of hexaethylbenzene dominated, and it was confirmed by NMR that this conformation persisted in solution [6].

The CSD was also mined for structures of 1,3,5-trimethylbenzene-based hosts, by using a similar search methodology and selection criteria to those described above. Of the total 194 crystal structures of such hosts found in the database, 88 (45.4%) were in a conformation defined as having all three binding elements on the same face of the central benzene ring. Overall, the proportion of triethylbenzene structures in their ideal “binding” conformation is lower than what would be expected based on the energy differences observed in solution (Table 1); however, it is significantly higher than the proportion of trimethylbenzene structures observed to be in the preorganized “binding” conformation, as expected.

### Computational analysis

Mislow originally calculated the energy of hexaethylbenzene in all possible conformations [6], and we started by repeating these calculations at the HF/6-31G* level of theory (Table 4). This method, although simple by modern standards, is suitable for conformational analysis and allowed us to calculate the relatively large systems (below) in a short time. Seven conformations including the lowest four conformations were studied and reported here. The results of the calculation in the gas phase were in good agreement with the values reported by Mislow (Table 4). The *ududud* conformer adopted the most stable conformation and the energy level of this conformation was 4.3 kcal/mol lower than the second most stable conformer, namely *uddudd*.

**Table 4:** Calculated energies for hexaethylbenzene conformations.

Conformation	Relative energy in gas phase (kcal/mol) (Mislow)	Relative energy in gas phase (kcal/mol) (this work)	Relative energy in water (kcal/mol) (this work)

*uuuuuu*	8.2	10.5	10.1
*uuuddd*	6.6	8.9	8.8
*uddddd*	5.9	8.7	7.5
*udduud*	3.7	4.4	4.4
*ududdd*	3.7	4.4	4.4
*uddudd*	3.5	4.3	4.3
*ududud*	0	0	0

One can expect a significant effect of the nature of the recognition elements on conformational energies. Many recognition elements that vary in shape, functionality, charge, and chirality have been reported. We picked pyrazole-derived hosts **4** (used for cation binding) [17] and imidazolium-based hosts **5** (used for anion binding) [18–19] as instructive systems for computational analysis (Figure 2). These hosts were chosen because (1) they are typical of the kinds of heterocycles often used as recognition elements in this family of hosts; (2) we wished to examine the effects of charge and solvation on conformational energies; and (3) they are nearly isosteric to each other, allowing us to separate out the influences of sterics and charges.

All calculations were carried out both in the gas phase and in the implicit water environment, as implemented in Spartan ’10 (SM8 model). These calculations were used to identify the global minimum-energy conformation for each host, and to determine the relative energies for each of the other conformations in each series (Table 5).

**Table 5:** Calculated energies for conformations of test hosts **4_Et_** and **5_Et_**.

Conformation	Host **4_Et_** relative energy in gas phase (kcal/mol)	Host **4_Et_** relative energy in water (kcal/mol)	Host **5_Et_** relative energy in gas phase (kcal/mol)	Host **5_Et_** relative energy in water (kcal/mol)

*uuuuuu*	12.4	10.4	11.7	9.9
*uuuddd*	9.9	9.9	4.3	6.2
*uddddd*	7.8	7.5	2.5	6.1
*udduud*	4.6	3.1	0.3	4.4
*ududdd*	4.6	4.3	0.4	4.0
*uddudd*	2.8	2.9	−0.1	3.8
*ududud*	0	0	0	0

Unlike simple hexaethylbenzene, the imidazolium groups of **5_Et_** provided a very different result when examined by gas-phase calculations. The conformers *ududud* and *uddudd*, are almost of the same energy and the next two most stable conformers, *ududdd* and *udduud*, lie only 0.3 and 0.4 kcal/mol above the *ududud* conformer. When the solvent condition is changed to water the trend of the results is much closer to those of hexaethylbenzene. The ideal *ududud* conformation is 3.8 kcal/mol lower than the second lowest conformation. No such change is observed for hexaethylbenzene itself on comparison of gas-phase and water calculations (Table 4). We expect that these differences arise from the overwhelming influence of (inadequately screened) charge–charge repulsion in the gas-phase calculations on **5_Et_**.

The calculated results of pyrazole-substituted **4_Et_** are quite similar in the gas phase and water. On comparison of the results of **4_Et_** to hexaethylbenzene, it is seen that the sequential ordering of the conformational energies is the same. But the *ududud* conformer of pyrazole-substituted **4_Et_** is only 2.9 kcal/mol more stable than the second-lowest-energy conformation in water, a gap that is a significant 1.4 kcal/mol smaller than the value calculated for hexaethylbenzene (4.3 kcal/mol). We interpret this difference in terms of the steric clashes between neighboring groups that occur in nonideal conformations such as *uddudd*. Planar, sp^2^-hybridized heterocycles on **4_Et_** have reduced steric demand relative to the sp^3^-hybridized CH_3_ groups that clash with neighboring substituents in hexaethylbenzene. These results show in general that the conformational-energy calculations for hexaethylbenzene cannot be simply applied to all 1,3,5-triethylbezene-based hosts. Although all the calculations in our study showed that *ududud* was the preferred conformation, the energy gap between the ideal conformer and the next most stable conformer depends strongly on the substituents.

The conformational energy landscapes of 1,3,5-trimethylbenzene-based hosts and their unsubstituted analogs are much simpler. There are only two possible conformations to be considered in these systems: “*uuu*,” in which all three substituted arms are directed toward the same face of the benzene, and “*uud*,” in which one binding arm is directed toward the opposite face of the benzene from the other two. Imidazolium-substituted hosts **5_Me_** and **5_H_** both show a preference for the nonideal *uud* conformation in the gas phase, which we can again attribute to the mutual repulsion of the positively charged substituents. This difference disappears when the calculation is carried out in water, in which the alike charges are more effectively screened from each other, and the *uuu* conformers are favored by 1.0 kcal/mol (for **5_H_**) and 0.5 kcal/mol (for **5_Me_**). The *uuu* conformers that are best suited for binding are favored for the pyrazole-substituted hosts **4_H_** and **4_Me_**, in the gas phase and in water, by values that range from 0.7–1.7 kcal/mol (Table 6). This result is not intuitive. The steric gearing that could possibly be provided by the methyl groups comes only from C–H bonds: A single C–H bond directed toward one face of the central benzene ring and two C–H bonds directed toward the other. Compared to the steric gearing provided by the ethyl groups, we assume that the magnitude of the possible energetic contribution to preorganization from the methyl groups is minor.

**Table 6:** Calculated energies for conformations of test hosts **4_Me_**, **4_H_**, **5_Me_**, and **5_H_**.

Conformation	Host **4_Me_** relative energy (kcal/mol)		Host **4_H_** relative energy (kcal/mol)		Host **5_Me_** relative energy (kcal/mol)		Host **5_H_** relative energy (kcal/mol)	
	gas	water	gas	water	gas	water	gas	water

*uud*	0.9	0.8	1.7	0.7	−1.8	0.5	−0.3	1.0
*uuu*	0	0	0	0	0	0	0	0

These calculations collectively show that, except in the case of exaggerated charge–charge repulsions present in the gas phase, all hosts of types **5_Et_**, **5_Me_**, and **5_H_** prefer the conformations in which all binding substituents are directed toward the same face of the central benzene ring. While a complete analysis would take all conformations (and their energies) into account, a simple and useful basis for evaluating these calculations of conformational energy differences is to compare the energy gaps between the lowest energy conformations and their next-highest congeners in each series, as these are the two conformations that would be most heavily populated in solution. The dependence of these gaps on the nature of the recognition substituents (imidazolium, pyrazole, or ethyl groups) is discussed above. But what about comparing the use of either ethyl or methyl groups as interposing or preorganizing elements for a given type of host? We calculate that the energetic preference for the “binding” conformation (defined as *ududud* for ethyl-substituted hosts and *uuu* for methyl-substituted hosts) is greater for ethyl-substituted hosts in general, being 3.8 kcal/mol for imidazolium **5_Et_**, (compared to 0.5 kcal/mol for imidazolium **5_Me_**) and 2.9 kcal/mol for pyrazole **4_Et_**, (compared to 0.8 kcal/mol for imidazolium **4_Me_**). This suggests that the steric gearing offered by the ethyl groups confers some energetic advantage over the methyl groups, but that the size of this advantage is dependent on the groups involved.

### Calculated dynamics and rotational barriers

**Figure 4:**
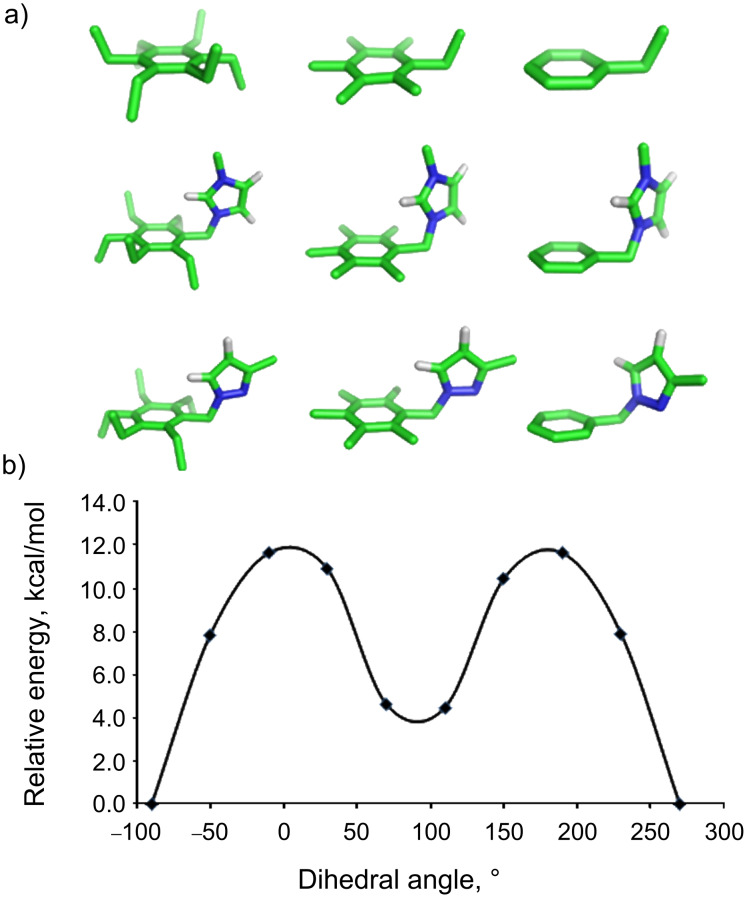
a) Structures used to calculate energy profiles at the starting geometry. b) An example of an energy profile arising from dihedral driving calculations on **1**_Et_. The smoothed line is intended to guide the reader’s eye.

Although kinetics has no bearing on binding thermodynamics, we sought also to understand computationally the dynamics of these different hosts. Molecular-dynamics simulations carried out at 300 K showed little or no dynamic exchange of conformations. Simulations carried out at the artificially elevated temperature of 400 K showed little more in the way of conformational exchange (one change of conformation for pyrazole host **4_Et_** and two for **4_Me_** during 10 ns). The unsubstituted host **4_H_** is a more mobile system, as indicated by the occurrence of 177 exchanges during the same simulation period. Faced with evidence that the barriers to exchange of “up” and “down” conformers in the sterically congested 1,3,5-triethylbenzene and 1,3,5-trimethylbenzene systems are too high to examine conveniently by MD simulations, we turned instead to a calculation of the barriers to bond rotation for a given set of substituents. These calculations were run on models composed of one of the test substituents (ethyl, pyrazolyl-CH_2_, or imidazolium-CH_2_) flanked by ethyl groups, methyl groups, or protons at the *ortho* and all other ring positions (Figure 4). The dihedral angle between the central benzene ring and the pendant substituent was constrained at 40° intervals between −180° and +180° and minimized at each stage in order to generate an energy profile for simple bond rotation for each type of host. This type of analysis ignores correlated bond rotations, which are sometimes important in sterically crowded systems. We make this assumption because Mislow’s original NMR studies demonstrated experimentally that there are no such correlated motions, even in highly crowded hexaethylbenzene [6]. Exemplary dihedral driving data, and the barriers thus calculated, are presented in Figure 4 and Table 7. The calculated rotation barriers for ethyl directed hosts are in the same range as previously reported values for related systems, which were determined by variable temperature NMR to be 9.3–11.8 kcal/mol [6,20].

**Table 7:** Calculated energy barriers (kcal/mol) to bond rotation, with respect to the rotating functional group and neighboring substituents.

	*ortho* substituents		
Rotating substituent	Et	Me	H

Et	11.6	9.0	4.3
Pyrazole-CH_2_	10.3	7.7	1.0
Imidazolium-CH_2_	15.7	9.5	3.5

As with the MD simulations, these results indicate that both ethyl and methyl *ortho* substituents cause high barriers to rotation (7.7–15.7 kcal/mol). Compared to the unsubstituted molecule, the ethyl *ortho* substituent provides 10.3–15.7 kcal/mol and the methyl *ortho* substituent provides 7.7–9.5 kcal/mol. We also find in this data a strong dependence on the nature of the rotating substituent, which is not easily explained by sterics. In general, the lowest barriers are calculated for pyrazole-CH_2_, while the nearly isosteric imidazolium-CH_2_ has significantly higher barriers across the board. In this result, again, we see that the rotation barriers offered by the ethyl groups confer some energetic advantage over the methyl groups, but the size of this advantage can be small and is dependent on the groups involved.

### A consideration of entropic effects

So how do these collective data inform us on the relative abilities of ethyl-, methyl-, and unsubstituted hosts to bind guests? The energies (*E*) calculated here are most closely akin to enthalpies (∆*H*), and neglect differences in entropy (∆*S*) from one host type to another. The aspects of host entropy that might contribute to guest binding, i.e., translational, vibrational, solvation, and configurational entropy, are worth separate discussions. Entropic effects arising from translation are not likely to depend strongly on host conformation (i.e., all conformations experience the same degree of reduced translational freedom upon binding), and we can assume that the presence of ethyl or methyl groups has little effect on translational entropy. We expect that changes in both vibrational entropy and solvent entropy will be highly variable for different systems in this class. While they might yield significant differences, their influence on binding energies cannot be predicted in a general way that depends on ethyl or methyl substitution. But the configurational entropy of “preorganized” systems like these is a fertile ground for discussion. In binding equilibria, the configurational entropy of a host is most frequently discussed in terms of the number of rotatable bonds (N_rot_) in free and bound states, which is a surrogate for considering the probability that a given conformation is occupied before and after binding [21–22]. Various schemes have been proposed for calculating the energetic contributions of these differences based on differences in N_rot_ between free and bound states; whatever the details of the calculations, a negative value for ∆N_rot_ upon binding (i.e., a transition to a more ordered state) is unfavorable. The unsubstituted hosts, such as **1_H_**, lose three rotatable bonds upon forming a host–guest complex (∆N_rot_ = −3), as do the methyl-substituted hosts such as **1_Me_**. Consideration of the ethyl-substituted hosts, such as **1_Et_**, becomes a bit tricky. If one considers that the system is perfectly fixed before and after binding then ∆N_rot_ is 0 (which is more favorable for binding). This kind of analysis was used by Raymond, in which it was estimated that the installation of ethyl groups produced a favorable *T*∆*S* of 4.5 kcal/mol (of the total of the 5.4 kcal/mol favorable binding energy). However, we have turned up no report that was published since with such a dramatic difference in overall binding energy. While host **3_Et_** has been shown to bind citrate in an entropy-driven manner, no comparison to **3_H_** or **3_Me_** was made, and the authors posit a significant role of solvent entropy in explaining their experimental data [23]. Overall, no specific measurement exists that correlates a large favorable change in entropy to the installation of ethyl groups.

Some insight is offered by the crystallographic survey we present above, which contains many structures for which either binding arms or ethyl groups (or both) are disordered. Our computational data suggests that these alternate conformations can be disfavored by small energies and may be significantly populated at room temperature (depending on the identity of the substituents). Further, the calculated bond rotation barriers for any of our ethyl-substituted model hosts are low enough that they would be easily surmounted at room temperature. It is interesting, therefore, to consider the possibility that, with a ∆N_rot_ of up to −6 (depending on the number of bonds free in unbound state and frozen in the bound state), some ethyl-substituted hosts may have an entropic disadvantage relative to methyl- and unsubstituted hosts. Given our analyses of existing data, it is likely that the true nature of the configurational entropic contributions lies somewhere between the two extremes. While entropic effects have surfaced in general in some classic studies [23], there is little or no experimental data on the separation of entropic contributions to host behavior in these systems, so this must remain, for now, a hypothesis awaiting experimental conformation.

## Conclusion

The picture that emerges from the combined surveys of crystallographic structures and binding affinities measured in solution is that the effect of installing ethyl or methyl groups onto supramolecular hosts is often favorable (as expected), but that the correlations between preorganized structures and binding affinities is nontrivial. Our computational data adds to this survey a basis for understanding the observed differences in energies that are most often invoked when discussing host preorganization, while also contributing additional evidence for variable behaviors that depend on the identities of molecular recognition elements and not purely on the scaffolds. The evidence collected here and elsewhere suggests that the installation of ethyl or methyl groups at 1,3,5 positions leads to consistent but relatively small increases in binding affinity relative to unsubstituted hosts. Given the overall variability we observe (and the desire of most researchers to synthesize only a single host for any given job), we suggest that carrying out the simple, broadly accessible calculations of the types described here may guide researchers in the selection of optimum substituents and scaffolds before synthesis begins.
